# Digital accessibility in the era of artificial intelligence—Bibliometric analysis and systematic review

**DOI:** 10.3389/frai.2024.1349668

**Published:** 2024-02-16

**Authors:** Khansa Chemnad, Achraf Othman

**Affiliations:** Mada Qatar Assistive Technology Center, Doha, Qatar

**Keywords:** digital accessibility, artificial intelligence (AI), research analysis, systematic review, persons with disabilities

## Abstract

**Introduction:**

Digital accessibility involves designing digital systems and services to enable access for individuals, including those with disabilities, including visual, auditory, motor, or cognitive impairments. Artificial intelligence (AI) has the potential to enhance accessibility for people with disabilities and improve their overall quality of life.

**Methods:**

This systematic review, covering academic articles from 2018 to 2023, focuses on AI applications for digital accessibility. Initially, 3,706 articles were screened from five scholarly databases—ACM Digital Library, IEEE Xplore, ScienceDirect, Scopus, and Springer.

**Results:**

The analysis narrowed down to 43 articles, presenting a classification framework based on applications, challenges, AI methodologies, and accessibility standards.

**Discussion:**

This research emphasizes the predominant focus on AI-driven digital accessibility for visual impairments, revealing a critical gap in addressing speech and hearing impairments, autism spectrum disorder, neurological disorders, and motor impairments. This highlights the need for a more balanced research distribution to ensure equitable support for all communities with disabilities. The study also pointed out a lack of adherence to accessibility standards in existing systems, stressing the urgency for a fundamental shift in designing solutions for people with disabilities. Overall, this research underscores the vital role of accessible AI in preventing exclusion and discrimination, urging a comprehensive approach to digital accessibility to cater to diverse disability needs.

## 1 Introduction

Digital accessibility is integral to modern times, especially because a significant percentage of the population is with one or multiple disabilities today. According to the World Health Organization (WHO), 16% of the global population (or ~1.3 billion people) currently suffers from significant disability (World Health Organization, 2022). This statistic highlights the need for digital content and services to be accessible to people with disabilities so that they are not excluded from actively participating the digital society today (Dobransky and Hargittai, 2016). Consequently, digital accessibility is not only a social responsibility, but also essential for legal compliance, inclusion, and business benefits. It ensures that everyone, regardless of their abilities or disabilities, has equal access to digital content and services, and is an essential factor of an organization that provides digital content or services. Digital accessibility, as defined by the Web Accessibility Initiative (WAI), implies that people with disabilities should be able to access, navigate, perceive, and interact with content [Initiative (WAI), 2022]. Digital accessibility refers to the practice of designing digital systems and services in a manner that makes them accessible to all individuals, including those with disabilities (Sharma et al., 2020). This includes ensuring that websites, software, and other digital products can be used by people with visual, auditory, motor, or cognitive impairments. Therefore, it is important to ensure that everyone, regardless of their ability, can access and use digital products without facing barriers or discrimination. If this is not done, it may result in people with disabilities being excluded from opportunities, diminishing their degree of independence.

Artificial intelligence (AI) is the capacity of a machine or computer system to simulate and perform tasks that typically require human intelligence, such as logical reasoning, learning, and problem resolution (Hassani et al., 2020). The determination of when applications and services can be classified as intelligent, as opposed to merely emulating intelligent behavior, is a topic of debate (Schank, 1991). Notwithstanding the ongoing philosophical discourse surrounding the extent and limitations of AI, remarkable progress has been made in a vast array of disciplines, such as machine learning, natural language processing, and computer vision. For example, AI-powered voice assistants such as Amazon Alexa, Apple Siri, and Google Assistant have become increasingly popular because of their ability to interpret natural language and offer user-friendly responses (McLean et al., 2021). In addition, AI pattern recognition algorithms have proven beneficial in several applications, including facial recognition, image processing, and object detection (Abiodun et al., 2019; Fu, 2019; Liu et al., 2020). AI has benefited industries by monitoring and detecting defects in various processes, resulting in increased productivity and decreased downtime (Pimenov et al., 2023). AI has also been instrumental in forecasting using intricate data analysis to make predictions. AI has been beneficial for improving treatment processes in the healthcare industry. For instance, tools powered by AI have been used to analyze medical images and identify abnormalities, resulting in more precise diagnosis and treatment recommendations (Liu et al., 2006). Smart assistive technologies, such as wheelchairs designed for people with limited mobility (Leaman and La, 2017) and canes intended for people with visual impairments (Hapsari et al., 2017), leverage advancements in AI to offer enhanced products and services. These advancements demonstrate the immense potential of AI in improving accessibility and user experience, making it an intriguing field of study for both researchers and practitioners.

The importance of digital accessibility in the AI era cannot be overstated. With the increasing use of AI in all spheres of life, it is crucial to ensure that these technologies are accessible to all individuals. This review presents the current state of the application of AI in the digital accessibility sector and proposes a classification system for identifying accessibility standards and frameworks, challenges, AI methodologies, and functionalities of AI in digital accessibility.

The next section provides background information on AI and its applications in various industries. Subsequently, an overview of current applications of AI in the digital accessibility sector is provided. This is followed by the research methodology, which describes the process of systematic review mapping and presents research results based on a classification framework. Subsequently, we discuss the current literature on the four dimensions of accessibility frameworks/standards, challenges, methodologies, and applications of AI for digital accessibility. Finally, future research directions and implications are discussed.

## 2 Background

As artificial intelligence continues to permeate various aspects of our lives, it is important to investigate its impact on the digital accessibility of people with disabilities. The inception of the term “Artificial Intelligence” or “AI” dates back to the 1950's when it emerged as a concept for designing machines with the ability to perform tasks that resemble human-like cognitive abilities (Schwendicke et al., 2020). In recent years, AI has made significant advancements in various industries including healthcare, finance, and transportation. With the development of machine learning, natural language processing, and deep learning algorithms, machines can now learn from data and improve their performances over time. This has the potential to significantly improve accessibility for people with disabilities, particularly in the health care industry. For example, AI is already being used to analyze medical images and diagnose diseases, such as cancer (Bi et al., 2019). In the finance industry, AI can also be used for tasks such as fraud detection and credit scoring (Zhou et al., 2018), which could potentially affect the financial accessibility of people with disabilities. However, it is important to monitor the use of AI in finance to ensure that it does not perpetuate discrimination against people with disabilities or other marginalized groups. Overall, although AI has the potential to improve accessibility and inclusion, it is important to approach its development and implementation with caution and focus on inclusivity.

Rapid advancements in technology have led to an increase in the use of digital devices for various purposes, including access to healthcare services. However, not everyone has equal access to these digital resources because of various barriers, such as physical, sensory, and cognitive disabilities. AI-powered technologies such as Automated Speech Recognition (ASR), Google Neural Machine Translation (GNMT), Google Vision API, and DeepMind are increasingly being used to improve accessibility for people with disabilities (Bragg et al., 2019). These technologies have the potential to transform interactions with digital devices and platforms. For example, ASR can provide captions and subtitles for video content (Alonzo et al., 2022), and image and facial recognition technologies can assist people with visual impairments (Feng et al., 2020). The utilization of AI-generated summaries in digital content can provide an advantage for screen reader users by breaking down lengthy texts into more manageable portions (Chen et al., 2023). Furthermore, AI can simulate user behavior to pinpoint and rectify navigation issues, and automate regression testing. Additionally, the implementation of AI-powered chatbots and virtual assistants can offer accessible communication options for people with speech and hearing impairments (Shezi and Ade-Ibijola, 2020; Subashini and Krishnaveni, 2021). However, the potential risks associated with AI in this context must be considered, such as the possibility of perpetuating existing biases and neglecting the needs and experiences of certain groups of people with disabilities. It is crucial to address these potential drawbacks to ensure that these technologies are truly inclusive and provide benefits for all users.

Machine learning algorithms can recognize patterns and identify accessibility barriers to digital content (Abduljabbar et al., 2019). For example, image recognition algorithms can automatically provide alternative text descriptions for images, making them accessible to the visually impaired (Bigham et al., 2006). Similarly, natural language processing algorithms can identify and correct language that may be difficult for people with cognitive impairments to understand (Le Glaz et al., 2021). By creating machine-learning algorithms that specifically address the issues of digital accessibility, we can create technology that benefits all users, regardless of their abilities. However, it is crucial that these algorithms are developed with inputs from people with disabilities to ensure that they are effective and meet their needs. In addition, it is important to consider the potential biases present in the data used to train these algorithms, as they may perpetuate existing inequalities and exclusion. By addressing these issues and prioritizing accessibility in machine-learning algorithms, we can work toward a more inclusive and equitable digital future for all.

Advancements in AI have created new opportunities to enhance digital accessibility for people with disabilities (Hapsari et al., 2017). However, as AI technology progresses, it is crucial to closely examine its impact on accessibility and to ensure that these technologies are developed in an equitable and inclusive manner. Despite the growing interest in the intersection of AI and digital accessibility, a comprehensive systematic review of the current state of knowledge and practices in this field is yet to be conducted. This systematic review aimed to fill this research gap by providing a comprehensive analysis of the current state of knowledge and practices related to AI and digital accessibility. By reviewing the existing literature, this study offers valuable insights into the potential benefits of AI for people with disabilities as well as identifying potential challenges and opportunities. Furthermore, this review can guide future research and development activities toward creating more inclusive and accessible technologies. The results of this systematic review can provide valuable resources for researchers, practitioners, and policymakers working in the fields of digital accessibility and AI by identifying gaps in current knowledge and practices, which can help promote digital accessibility and support the development of inclusive technologies that benefit everyone.

## 3 Materials and methods

**Figure 1:**
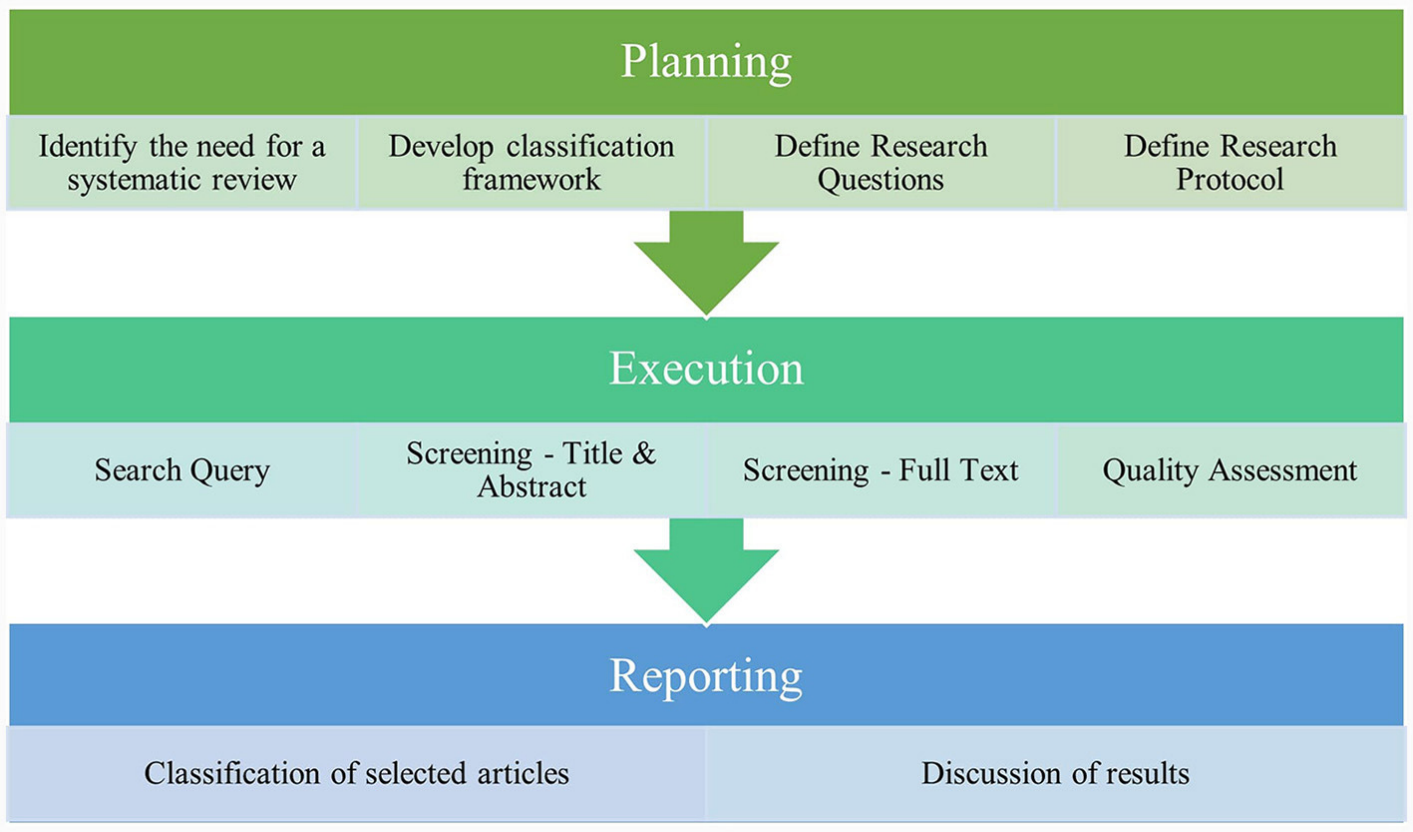
Phases of the systematic review.

This literature review followed the procedures and processes for conducting literature searches proposed by Watson (2015), and was conducted in three phases: planning, execution, and reporting. The systematic review methodology used in this study incorporated the strategies and guidelines outlined by Kitchenham et al. (2007) and Ali et al. (2018, 2020, 2021, 2023). Figure 1 depicts the steps involved in the systematic review.

In the planning phase, the need for a systematic review was identified, a classification framework was defined, research questions were defined, and a research protocol was developed. During the execution phase, the search query was conducted based on the following keywords: exclusion and inclusion criteria were applied; screening based on title and abstract reading, full-text reading, and quality assessment were conducted. The reporting phase included classification of the chosen articles and discussion of the findings.

### 3.1 Planning stage

#### 3.1.1 Identify the need for a systematic review

To our knowledge, no systematic review has comprehensively outlined these research findings, while providing a profound analysis of the research and practice related to the topic of digital accessibility, with a specific focus on AI applications for people with disabilities. Although limited in number, existing surveys on digital accessibility are often specialized in nature, with many being country-specific studies or tailored to specific fields, such as education and software engineering processes (Bong and Chen, 2021; Chadli et al., 2021; Paiva et al., 2021; Prado et al., 2023). However, despite these valuable efforts, no comprehensive systematic review has yet extensively examined the intersection of digital accessibility and AI applications within the existing literature. This study seeks to address this gap by providing an extensive analysis of this field, revealing potential benefits, challenges, and opportunities.

#### 3.1.2 Develop a research review protocol

In this step, the research review protocol used for the literature review was finalized. The classification framework is based on a model initially developed by Ngai and Wat (2002), which is based on how advanced technologies have enhanced various fields. The framework was developed and modified by Ali et al. (2018, 2020, 2021, 2023). The framework used in our study takes into consideration four dimensions of how AI is used in different sectors about accessibility: applications, AI methodology and techniques, design standards and frameworks used, and challenges. Table 1 provides more details on the classification framework used in our study.

**Table 1:** Classification framework (modified from Ngai and Wat, 2002).

**Applications**	**AI methodology**	**Design standards/guidelines/frameworks**	**Challenges**
• Artificial vision • Navigation • Fire safety • Road safety • Educational • Banking • lighting solutions • Virtual assistant • Communication with smart devices • Sign language recognition • Accessible transport • Entertainment	• Computer vision • Edge AI • NLP • Machine learning • Deep learning	• Design standards • Accessibility standards	• Data challenges • Technical issues • Operational challenges • Knowledge and awareness • Security and privacy

#### 3.1.3 Defining the research questions

Formulating the research questions for this study is a crucial step in systematic reviews (Paul et al., 2021). The research questions identified for the study are as follows:

RQ 1. What are the AI applications that have been used to enhance digital accessibility?
RQ 2. What are the different AI techniques that have been used to enhance the accessibility of digital systems for people with disabilities?
RQ 3. What are the key challenges and barriers that must be overcome to achieve accessible AI systems for people with disabilities ?
RQ 4. What are the different accessibility standards that have been used to guide the design and implementation of accessible AI systems?

#### 3.1.4 Defining the strategies for article selection

To minimize bias, strategies for article selection were determined at this stage. As a part of this process, a comprehensive search strategy was created to encompass a wide-ranging search of various databases, and a manual review of the selected articles was conducted. The databases chosen for this systematic review included the ACM Digital Library, IEEEXplore, ScienceDirect, Scopus, and Springer. During the research process, filtering tools were employed for every database to minimize duplication. In conducting the manual review, the broad manual review method was utilized, which entailed scanning the title and abstract of each research article (Golder et al., 2014), followed by reading the full content of the selected articles to eliminate any irrelevant ones. Table 2 lists the eligibility criteria used in this study.

**Table 2:** Eligibility criteria for article inclusion in the review.

**Inclusion criteria**	**Exclusion criteria**
Papers published between the years 2018–2023	Papers not written in English.
The paper should have been written in English	Duplicated studies
The paper should answer one or more of the research questions	Papers that were not peer-reviewed i.e., reviews, books, posters, and editorials;
The publication should have been peer reviewed and should either be a journal article or a conference paper.	The paper is about elderly or medical diagnosis
The paper length shall be a minimum of five pages excluding references.	Paper is not about digital accessibility

### 3.2 Execution stage

The execution stage involved the implementation of the steps outlined in the planning phase. The main steps undertaken in the execution stage are as follows:

- To ensure a thorough search, we utilized key terms extracted from relevant research papers and research questions, including synonyms, acronyms, and spelling variations of these terms. The search terms were then classified into three categories: those related to AI, technology-specific terms, and accessibility in the context of disability. The keywords identified for this study were: ((“Artificial Intelligence” OR “AI”) AND (“Digital” OR “technology” OR “technologies” OR “technological”) AND (“accessibility” OR “inclusion” OR “inclusivity” OR “disability” OR “disabled” OR “special needs” OR “impaired” OR “impairment”)).
- Filtering tools were used to refine the research results while conducting the online database search. Several filters were implemented in this study, including the year of publication (2018–2023), document type (journal articles and conference papers), and language (English). Given the vast quantity of results, additional filters, such as “title,” “abstract,” and “keyword” search, as well as “open access” filters, were utilized to refine the search. Of the 50,655 search results returned by the Springer database, only the first 1,000 were considered for this review.
- The articles were subjected to abstract and keyword screening followed by full-text screening.
- To ensure the validity of the articles included in this study, certain quality assessment criteria were applied to confirm their value. Quality assessment of the candidate papers followed the guidelines outlined in Kitchenham et al. (2007) study. Consequently, we formulated the following questions to evaluate quality.

1. Are the aims clearly stated?
2. Are data collection methods adequately described?
3. Are all of the study questions answered?
4. Is the AI technique and methodology used in this study fully defined?
5. How do these results add to the literature?
6. Are the limitations of this study were adequately addressed.
7. How clear and coherent are reports?

Each QA inquiry was evaluated using a three-point rating system. A response of “yes” received a score of 1, a “partial” response earned 0.5 points, and a “no” response was assigned a score of 0. If the study effectively addressed the QA question, it received a full point, whereas a partial response warranted 0.5 points. Any paper that failed to address the QA question was assigned a score of 0. The quality of the research articles was assessed using the QA questions to determine the overall quality score for each study. An inclusion criterion was established, requiring a minimum overall score of four for research inclusion. Studies with scores below four were excluded.

### 3.3 Summarizing stage

**Figure 2:**
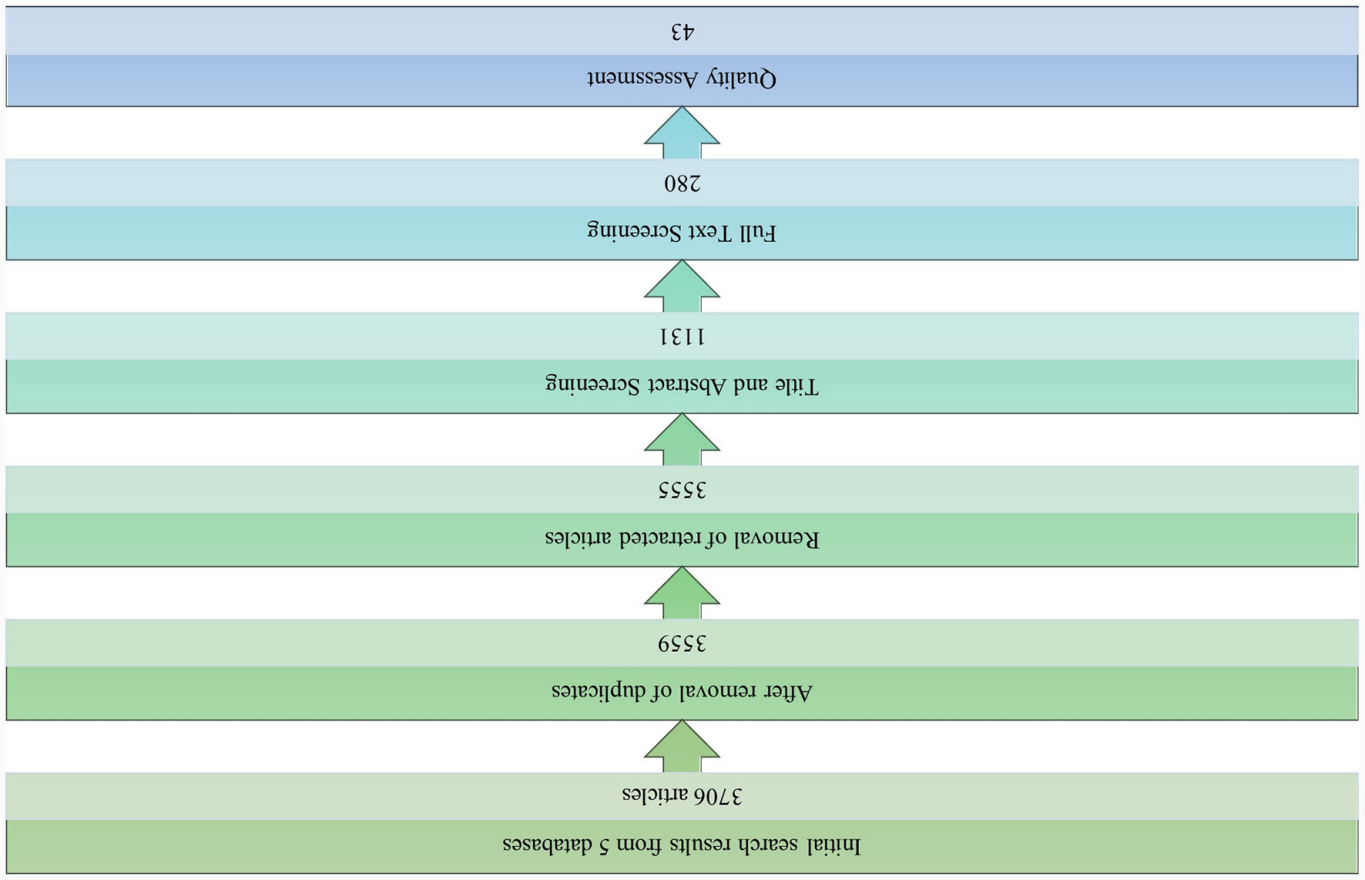
Review search results.

Data collection for this review was conducted between May 18, 2023, and May 24, 2023. Figure 2 shows the final number of articles selected for this review. The search results based on the keywords and search filters applied identified 3,706 articles. Duplicates and retracted articles were removed prior to the abstract, title, and keyword screening. In total, 2,424 articles were removed at this stage. During the full-text screening stage, 850 articles were excluded, leaving 280 articles. These articles were subjected to quality assessment, and 42 articles were retained for the final analysis.

## 4 Results and discussion

### 4.1 Distribution of article by publication year

**Figure 3:**
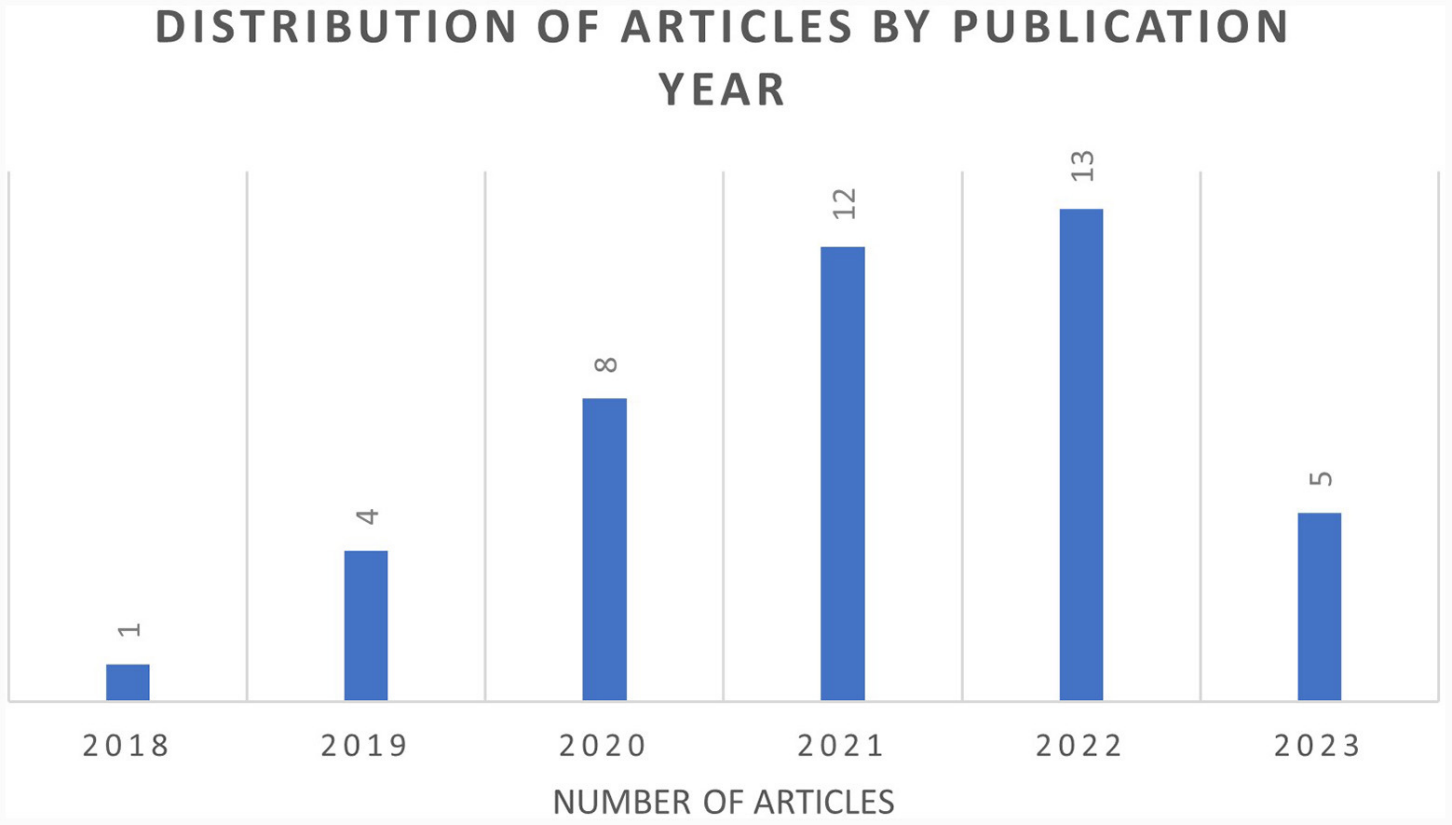
Distribution of articles by year.

Figure 3 illustrates the dissemination of articles by the year of publication. The first publication on the utilization of AI in accessibility was published in 2018, whereas the most substantial number of articles (13) were released in 2022. Conversely, only one article was published in 2018.

### 4.2 Distribution of articles by database

**Figure 4:**
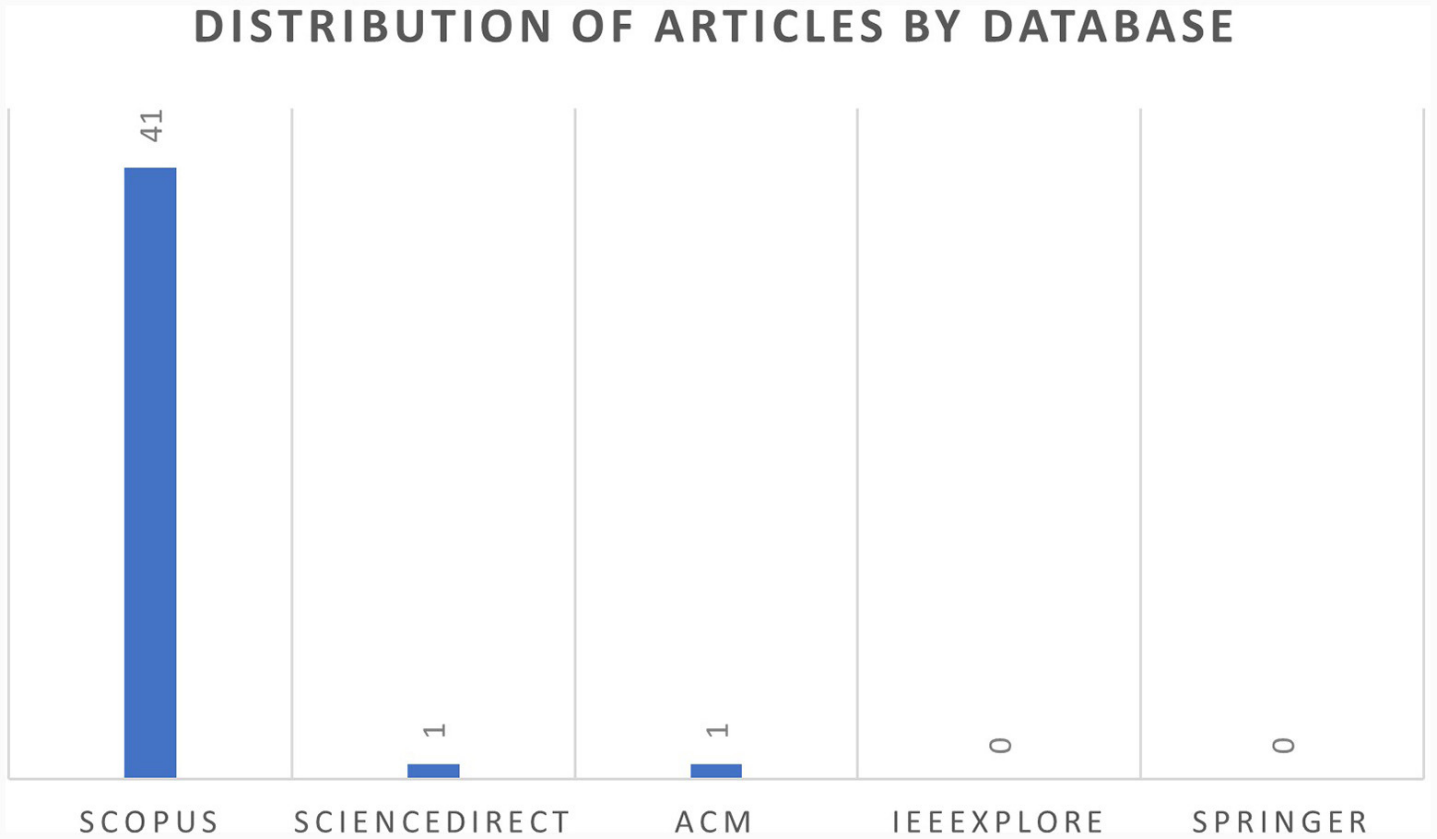
Distribution of articles by database.

Figure 4 depicts the distribution of the selected articles by the database source. A total of 41 articles were identified in Scopus, followed by one article each from ScienceDirect and ACM Digital databases. No articles were selected from IEEEXplore or Springer database, as they were either duplicates or had to be excluded as they did not meet the inclusion criteria during the screening process.

### 4.3 Research classification framework

The findings of the review were the result of a comprehensive analysis of articles related to AI and digital accessibility that were presented and evaluated. The classification framework was applied by considering four dimensions: accessibility guidelines, frameworks, standards, challenges, methodologies, and applications. Additional information is provided in Tables 3–6.

**Table 3:** Applications that have been developed to enhance digital accessibility.

**Category**	**Application**	**References**
Visual impairment	Provide an artificial vision for a perfect human vision	Balakrishnan et al., 2023
	Supporting visually impaired people during their navigation	Balakrishnan et al., 2023
	Virtual assistant	Royal et al., 2023
	Real-Time Fire Warning System	Abdusalomov et al., 2022
	Explores the shared privacy and ethical concerns of people with visual impairments	Akter et al., 2022
	Assist to perceive the world using a tactile glove and a webcam	Li et al., 2022
	Visualize environment with the help of handheld devices such as Mobile Phones	Chaitra et al., 2022
	Assist visually impaired pedestrians in safely crossing the street	Montanha et al., 2022
	Create a dataset for teachable object recognition with people who are blind or have low vision	Theodorou et al., 2021
	Automate the generation of tactile educational materials for enhancing educational material development for visually impaired and blind students	See and Advincula, 2021
	Enhance the accessibility of social media content for people with disabilities, particularly those with visual impairments	Duarte et al., 2021
	Mobile bank note recognition application that can help visually impaired people identify different bank note values	Thomas and Meehan, 2021
	Provide braille-readable captions for images to help visually impaired individuals better understand their surroundings and the images they encounter in the digital environment	Wadhwa et al., 2021
	System explicitly designed for visually impaired people for indoor and outdoor localization and navigation.	Lo Valvo et al., 2021
	Provide a multi-language interactive device for visually impaired persons to read and translate text from books, magazines, newspapers, and other printed materials	Harum et al., 2021
	Provide a solution to the problem of current lighting systems not providing necessary lighting comfort for visually impaired and elderly people	Karyono et al., 2020
	Provide walking path support for visually impaired university students	Kose and Vasant, 2020
	Conduct face-to-face video communication freely and confidently	Guo et al., 2021
	Provide an interactive learning experience for visually impaired children aged 6–14 years	Jayawardena et al., 2019
	Provide AI fully automated assistive technique for visually impaired people to perceive objects in surrounding and provide obstacle-aware navigation, where auditory inputs are given to users in real-time	Joshi et al., 2020
	AI-Vision smart glasses with 2 key functionalities: facial expression/age/gender recognition for meetings and color recognition for outfit sorting	Alashkar et al., 2020
	Help blind athletes' mobility and orientation, empowering them to run without a guide	Lucibello and Rotondi, 2019
	Provides near-real-time information about user's surroundings using a smartphone camera to help users navigate more effectively	Saha et al., 2019
	Provide students with visual impairments a virtual assistant in the lab that can be controlled using natural language	Watters et al., 2020
Speech and hearing impairment	Recognize the complex sign language	Ullah et al., 2023
	Offers highly accurate speech recognition and voice reproduction capabilities	Yang et al., 2023
	Automatic recognition of two-handed signs in Indian Sign Language (ISL) serving as teaching assistant	Sreemathy et al., 2022
	Aid people who are deaf or hard-of-hearing in communicating with their smart home devices	Rajasekhar and Panday, 2022
	Enhance information accessibility for Deaf and Hard of Hearing individuals by developing more effective American Sign Language (ASL) animations.	Al-Khazraji et al., 2021
	Provide real-time video transcripts for people in Saudi Arabia who are deaf or have hearing loss	Zaid alahmadi and Alsulami, 2020
	Conduct face-to-face video communication freely and confidently	Guo et al., 2021
	Harness virtual reality (VR) technology to enhance speech comprehension for individuals who are deaf or hard of hearing (DHH) and apply it to live communication during theatrical performances	Teófilo et al., 2018
	Aid students with autism spectrum disorder (ASD) in learning to code with the aid of an AI Companion (AIC)	Hughes et al., 2022
	Conduct face-to-face video communication freely and confidently	Guo et al., 2021
	Recognize the complex sign language	Ullah et al., 2023
Motor impairment	Increase the accessibility of digital games for players with motor impairments	Cimolino et al., 2021
	Provide cooperative traffic signal assistance for non-motorized users and disabilities	Yang et al., 2022
	Provide accessible bus rides for persons in wheelchairs	Lin et al., 2019
Neurological impairment/disorder	Improve accessibility and inclusion for dyslexic students in the learning system	Zingoni et al., 2021

**Table 4:** AI methodologies utilized to enhance digital accessibility.

**Category**	**AI techniques**	**References**
Edge AI		Yang et al., 2022
Computer vision	Instance segmentation	See and Advincula, 2021
	Object detection	Joshi et al., 2020; Lo Valvo et al., 2021; See and Advincula, 2021; Thomas and Meehan, 2021; Chaitra et al., 2022; Yang et al., 2022; Balakrishnan et al., 2023; Royal et al., 2023
	Image/object recognition	Jayawardena et al., 2019; Lin et al., 2019; Saha et al., 2019; Duarte et al., 2021; Abdusalomov et al., 2022
	Image classification	Thomas and Meehan, 2021
	Optical character recognition/text recognition	Duarte et al., 2021; Ingavelez-Guerra et al., 2022; Royal et al., 2023
Machine learning		Lin et al., 2019; Lucibello and Rotondi, 2019; Alashkar et al., 2020; Kosiedowski et al., 2020; Zingoni et al., 2021; Ullah et al., 2023
Deep learning		Lin et al., 2019; Karyono et al., 2020; Guo et al., 2021; Zingoni et al., 2021; Li et al., 2022; Montanha et al., 2022; Sreemathy et al., 2022; Ullah et al., 2023; Yang et al., 2023
	CNN	Jayawardena et al., 2019; Alashkar et al., 2020; Joshi et al., 2020; Lo Valvo et al., 2021; Thomas and Meehan, 2021; Wadhwa et al., 2021; Abdusalomov et al., 2022; Chaitra et al., 2022; Ingavelez-Guerra et al., 2022; Rajasekhar and Panday, 2022; Yang et al., 2022
	RNN	Lu et al., 2020; Wadhwa et al., 2021
NLP		Saha et al., 2019; Ingavelez-Guerra et al., 2022
	Text-to-speech	Jayawardena et al., 2019; Alashkar et al., 2020; Joshi et al., 2020; Harum et al., 2021; Abdusalomov et al., 2022; Royal et al., 2023
	Facial expression recognition	Hughes et al., 2022
	Speech recognition	Teófilo et al., 2018; Kose and Vasant, 2020; Zaid alahmadi and Alsulami, 2020; Efanov et al., 2022; Ingavelez-Guerra et al., 2022; Royal et al., 2023; Yang et al., 2023

**Table 5:** Digital accessibility frameworks.

**Category**	**Frameworks/standards**	**References**
Design standards	Framework for value co-design and co-creation	Vieira et al., 2022
	Universal Design for Learning (UDL) principles	Hughes et al., 2022; Ingavelez-Guerra et al., 2022
Accessibility standards	accessibility compliance testing (ACT) rules recommended by the World Wide Web Consortium (W3C)	Montanha et al., 2022
	Web Content Accessibility Guidelines (WCAG) 2.1	Acosta-Vargas et al., 2022
	ISO/IEC 25010:2011 Systems and software engineering	See and Advincula, 2021
	Universal design	Cimolino et al., 2021
	Player balancing	Cimolino et al., 2021
	Interface adaptation	Cimolino et al., 2021
	Digital Accessible Information System (DAISY) Standard	Harum et al., 2021
	CIE 1,231,997	Karyono et al., 2020
	CIE 196:2011	Karyono et al., 2020
	CIE 227:2017	Karyono et al., 2020
	Fermatean fuzzy information based decision-making framework	Hezam et al., 2023
	Guidelines for designing mobile apps for people who are deaf or hard of hearing (Yeratziotis and Van Greunen, 2013)	Zaid alahmadi and Alsulami, 2020
	Yue and Zin (2013) proposed a model based on Pugh's product development process (PDP) model	Zaid alahmadi and Alsulami, 2020

**Table 6:** AI enabled digital accessibility challenges.

**Category**	**Type**	**References**
Data challenges	Lack of data/challenges in data collection process	Lu et al., 2020; Park et al., 2021; Yang et al., 2022
	Data management and security	Vieira et al., 2022
	Data bias and discrimination	Zaid alahmadi and Alsulami, 2020; Guo et al., 2021; Theodorou et al., 2021; Vieira et al., 2022
Technical issues	Complexity	Lu et al., 2020; Cimolino et al., 2021; Theodorou et al., 2021; Li et al., 2022; Vieira et al., 2022; Ullah et al., 2023
	Lack of technical support/technical issues	Teófilo et al., 2018; Guo et al., 2021; Vieira et al., 2022; Hezam et al., 2023
	Fallibility of AI	Akter et al., 2022
	Hardware constraints	Rajasekhar and Panday, 2022
Operational challenges	Lack of infrastructure	Hezam et al., 2023
	Cost	Jayawardena et al., 2019; Karyono et al., 2020; Lo Valvo et al., 2021; Li et al., 2022; Vieira et al., 2022; Hezam et al., 2023
	Lack of implementation of accessibility regulations and standards	Karyono et al., 2020; Guo et al., 2021; Lo Valvo et al., 2021; Acosta-Vargas et al., 2022; Ingavelez-Guerra et al., 2022; Hezam et al., 2023
Knowledge and awareness	Lack of awareness	Karyono et al., 2020; Vieira et al., 2022; Hezam et al., 2023
Security and privacy	Privacy and surveillance	Akter et al., 2022; Hughes et al., 2022; Li et al., 2022; Vieira et al., 2022
	Security	Vieira et al., 2022

#### 4.3.1 (RQ 1) Dimension: disability types

##### 4.3.1.1 AI for the visually impaired

The reviewed literature emphasizes the development of various applications aimed at enhancing the mobility, safety, and quality of life of the visually impaired. These applications include an array of functionalities such as obstacle detection, navigation, educational support, and social media accessibility.

Several systems have been specifically designed for obstacle detection and spatial information. For example, Balakrishnan et al. (2023) presented a novel synchronization protocol for vision sensors that provides real-time obstacle detection and spatial detail to improve mobility and safety. Building upon this, Royal et al. (2023) went a step further by not only detecting objects but also offering real-time object recognition and text reading within images, thereby improving overall accessibility.

In the domain of safety, Abdusalomov et al. (2022) utilized AI-based fire detection and classification methods to monitor and forecast fire-related scenarios, thereby providing early warnings that are specifically tailored to the needs of blind and visually impaired individuals. Akter et al. (2022) examined shared ethical and privacy considerations relevant to people with visual impairments, making a significant contribution to the ethical development of assistive technologies.

The AviPer system utilizes a visual-tactile multimodal attention network that incorporates a self-developed flexible tactile glove and webcam (Li et al., 2022). This novel approach enables visually impaired individuals to perceive and interact with their surroundings. Furthermore, Chaitra et al. (2022) presented a viable and cost-effective solution for visualizing environments using handheld devices, such as mobile phones.

In urban environments, Montanha et al. (2022) employed context awareness and AI methods to aid visually impaired pedestrians in safely navigating roadways and generating real-time instructions based on traffic conditions.

Duarte et al. (2021) worked to enhance the accessibility of social media content, particularly benefiting people with visual impairments. In addition, Thomas and Meehan (2021) introduced a mobile banknote recognition application and Wadhwa et al. (2021) improved environmental understanding by offering braille-readable captions for digital images.

Lo Valvo et al. (2021) concentrated on the development of indoor and outdoor localization and navigation technologies for visually impaired individuals. Harum et al. (2021) presented a multi-language interactive device that enables the reading and translation of text from various printed materials. Karyono et al. (2020) addressed the issue of lighting discomfort experienced by the visually impaired and elderly, by introducing a specially designed system.

Kose and Vasant (2020) offered effective walking-path support to university students with visual impairments, enhancing their overall campus experience. Joshi et al. (2020) introduced an AI-based fully automated assistive system with real-time perception, obstacle-aware navigation, and auditory feedback. The AI-Vision smart glasses, outlined in Alashkar et al. (2020), utilize machine learning algorithms for facial expression/age/gender recognition and color recognition, providing assistance in daily tasks. Furthermore, See and Advincula (2021) automated the production of tactile educational materials, such as tactile flashcards, tactile maps, and tactile peg puzzles, which incorporate interactive tactile graphics and braille captions, serving as a versatile tool for teaching concepts such as shapes and geography to visually impaired and blind students.

Lucibello and Rotondi (2019), Saha et al. (2019), and Watters et al. (2020) aimed to improve the lives of both blind and visually impaired individuals. Lucibello and Rotondi (2019) focused on supporting blind athletes in the track and field, promoting independent running, and enhancing spatial awareness through the learning of echolocation skills. Saha et al. (2019) has created a system that provides near-real-time information about the surroundings through a smartphone camera, enhancing the mobility skills of people with visual impairments. Finally, Watters et al. (2020) aimed to provide visually impaired students with a virtual assistant in the laboratory that could be controlled through natural language, eliminating the need for specific keywords or phrases.

##### 4.3.1.2 AI for speech and hearing impairment

This section explores innovative technologies developed to cater to people with speech and hearing impairments. These technologies have been categorized into distinct applications that offer a range of benefits. For instance, a graphene-based wearable artificial throat was developed to provide highly accurate speech recognition and voice reproduction capabilities, particularly for those who have undergone laryngectomy (Yang et al., 2023). Additionally, a platform that recognizes complex sign language was designed to offer an effective means of communication for speech-impaired children (Ullah et al., 2023). The automatic recognition of two-handed signs in Indian Sign Language also serves as a teaching assistant, enhancing cognitive abilities and fostering interest in learning for hearing and speech-impaired children (Sreemathy et al., 2022). The real-time interpretation and recognition of American Sign Language contributes to seamless control of smart home components, addressing the specific needs of people who are deaf or hard-of-hearing in their interactions with smart home devices (Rajasekhar and Panday, 2022).

AI Technology has also been utilized to enhance information accessibility through more efficient American Sign Language animations (Al-Khazraji et al., 2021). Real-time video transcripts are provided to improve the accessibility of video content for people with hearing impairments or deafness in Saudi Arabia (Zaid alahmadi and Alsulami, 2020). Finally, the utilization of virtual reality technology in live communication during theatrical performances demonstrates its potential to enhance speech comprehension for people who are deaf or hard of hearing (Teófilo et al., 2018). These insights collectively highlight the diverse applications of AI in addressing the communication and accessibility needs of people with speech and hearing impairments, emphasizing the potential for transformative effects on their daily lives and societal inclusion.

##### 4.3.1.3 AI for autism spectrum disorder

Hughes et al. (2022) concentrated on advancements in virtual AI companion (AIC) and facilitating students with autism spectrum disorder (ASD) in STEM learning environments while fostering their social and communication skills.

##### 4.3.1.4 AI for neurological disorder

Zingoni et al. (2021) aimed to enhance accessibility and inclusion for dyslexic students by creating a supportive platform that utilizes best practices for educators and institutions to predict the most suitable methodologies and digital tools for students with the goal of addressing the challenges they face in their academic journeys.

##### 4.3.1.5 AI for motor impaired

Cimolino et al. (2021) aimed to tackle the issue of inaccessibility in gaming for people with disabilities, particularly those with spinal cord injuries, by proposing a novel method called “partial automation” that enables an AI partner to manage inaccessible game inputs to improve overall accessibility in gaming.

##### 4.3.1.6 AI for other disabilities

This section encompasses an array of innovative applications that cater to people with other disabilities. Campomanes-Alvarez and Rosario Campomanes-Alvarez (2021) focused on individuals with profound intellectual and multiple disabilities, devising the INSENSION platform to recognize facial expressions, thereby enabling interaction with digital applications and enhancing their quality of life. Second, Ingavelez-Guerra et al. (2022) implemented a multilevel methodological approach to automatically adapt open educational resources within e-learning environments, considering the diverse needs and preferences of students with various disabilities, including hearing impairments, physical disabilities, intellectual and developmental disabilities, visual impairments, and mental health issues. Third, Guo et al. (2021) presented a virtual human social system that empowered individuals with facial disabilities, deafmutes, and autism to engage in face-to-face video communication. Additionally, Hezam et al. (2023) utilized a hybrid multi-criteria decision-making method to prioritize digital technologies for enhancing transportation accessibility for people with disabilities, addressing barriers, and selecting technologies through an uncertain decision-making framework. Furthermore, Lu et al. (2020) aimed to aid people with disabilities in operating tractors, ensuring safe operation when consciousness and limb movements are inconsistent, whereas Lin et al. (2019) focused on providing wheelchair-accessible bus rides for people with disabilities. The INSENSION Platform for Personalized Assistance of Non-symbolic Interaction for individuals with profound intellectual and multiple disabilities (PIMD), developed by Kosiedowski et al. (2020), supports independence by recognizing non-symbolic behaviors and collecting contextual information through video, audio, and sensor data. Park et al. (2021) proposed an online infrastructure to enable large-scale remote data contributions from disability communities to create inclusive AI systems. These diverse applications underscore the importance of tailored technological solutions in promoting independence, accessibility, and improved quality of life for people with disabilities, emphasizing the need for an inclusive design and comprehensive consideration of user needs in the development of assistive technologies.

#### 4.3.2 (RQ 2) Dimension: methodology

The accessibility of digital systems for people with disabilities can be enhanced by employing various AI techniques, which can be classified into several domains including Machine Learning (ML), Deep Learning (DL), Natural Language Processing (NLP), Edge AI, and Computer Vision.

##### 4.3.2.1 Computer vision

Several studies have demonstrated diverse applications of computer vision in enhancing accessibility. Balakrishnan et al. (2023) utilized object recognition for identifying and predicting object types, employing techniques such as object localization and detection. Jayawardena et al. (2019) used computer vision for object recognition and hand gesture/movement recognition in an educational context. In addition, Lo Valvo et al. (2021) employed Convolutional Neural Networks (CNNs) for recognizing objects or buildings. Royal et al. (2023) developed a real-time object recognition and text extraction from images using deep learning algorithms in combination with the Pytesseract OCR Engine. Chaitra et al. (2022) employed a pre-trained Caffe Object Detection method to facilitate the text-to-signal processing feature summarizing the objects detected in the form of an audio catalog. Abdusalomov et al. (2022) employed AI techniques such as fire, object, and text recognition, along with object mapping. Duarte et al. (2021) employed various AI methodologies including image recognition, text recognition in images, semantic similarity measures between text descriptions and image concepts, and language identification. Saha et al. (2019) utilized AI techniques, including object recognition, within the Landmark AI computer vision system, which employed a smartphone camera to offer real-time information and identify and describe landmarks and signs in the user's surroundings.

##### 4.3.2.2 Natural language processing (NLP)

NLP plays a critical role in making digital systems more accessible. Ingavelez-Guerra et al. (2022) transformed digital educational resources using NLP for automatic speech recognition (ASR) to extract textual information from videos. In addition, Wadhwa et al. (2021) utilized NLP to preprocess raw image and caption data, incorporating a pretrained CNN for image feature encoding and an LSTM-based RNN for generating natural language descriptions. Google's speech-to-text recognition, which falls under NLP, was integrated to transcribe and process the spoken language into text for analysis (Zaid alahmadi and Alsulami, 2020). Furthermore, Joshi et al. (2020) implemented various NLP techniques including YOLO-v3 object detection, an optical character recognizer, and a text-to-speech module for generating audio prompts. Teófilo et al. (2018) combined AI techniques, including a speech-to-text algorithm for Portuguese, a sentence prediction algorithm for selecting the correct speech based on initial text, and a word correction algorithm to ensure the converted words are valid in the Portuguese language. Harum et al. (2021) leveraged third party apps such as Google Cloud services, including the Cloud Vision API for image-to-text conversion, the Cloud Translation API for translating the converted text, and Google Cloud Text-to-Speech for converting the translated text into speech.

##### 4.3.2.3 Edge AI

The system proposed by Yang et al. (2022) showcased the utilization of Edge AI in a cost-effective manner. The combination of Edge AI and computer vision enabled the system to employ one-stage detectors and 2-D human pose estimation for non-motorized users, emphasizing the importance of edge computing in efficient real-time processing of data. Additionally, the SINAPSI device in Lucibello and Rotondi (2019) employed Edge AI, utilizing ultrasonic sensors and machine learning algorithms for 3D environmental awareness, demonstrating its application in innovative environmental awareness solutions.

##### 4.3.2.4 Machine learning

Various studies have employed machine learning techniques to address accessibility challenges. For instance, Ullah et al. (2023) utilized KNN, decision trees, random forest, and neural networks to decipher sign languages, underscoring the versatility of machine learning in linguistic contexts. A range of machine learning techniques, including Naïve Bayes, K-Nearest Neighbor, Stochastic Gradient Descent, Logistic Regression, Neural Networks, and Random Forest, have been used to classify facial expressions and jaw movements (Campomanes-Alvarez and Rosario Campomanes-Alvarez, 2021). Zingoni et al. (2021) primarily employed machine learning techniques, starting with supervised ML algorithms, to predict appropriate supporting materials for dyslexic students based on questionnaire and clinical report data, with a potential transition to deep learning methods for more complex data processing. The Speech Recognition Engine in Jayawardena et al. (2019) utilized ML to recognize a user's voice commands, demonstrating the adaptability of machine learning across diverse applications.

##### 4.3.2.5 Deep learning

The utilization of deep-learning methodologies has been prevalent in several studies, including Royal et al. (2023), which facilitated real-time object recognition and text extraction using deep-learning algorithms. Li et al. (2022) used a visual-tactile fusion classification model, which is a multimodal deep learning model that combines visual and tactile information to classify objects, and three attention mechanisms, namely temporal, channel-wise, and spatial attention, which are used to improve the accuracy of the classification model. See and Advincula (2021) utilized a model based on the Mask RCNN model trained using the common objects with context (COCO) dataset with a backbone of ResNet-50.

Rajasekhar and Panday (2022) implemented a 1D CNN as the deep learning model in an ASL gesture interpreter, while Thomas and Meehan (2021) employed a CNN to implement object detection in a banknote recognition system.

Zingoni et al. (2021) primarily employed machine learning techniques, starting with supervised ML algorithms with a potential transition to deep learning methods for more complex data processing. Kosiedowski et al. (2020) harnessed video and audio analysis, pattern recognition, and deep-learning techniques to recognize various aspects of users with Profound and Multiple Intellectual Disabilities. The integration of YOLOv3, a real-time image recognition model, by Lin et al. (2019) highlighted the effective use of deep learning for image recognition and notification. Yang et al. (2023) utilized spectrogram-based feature extraction with pre-trained neural networks, k-fold cross-validation, and an ensemble model involving AlexNet, ReliefF, and an SVM classifier to enhance speech recognition accuracy. Abdusalomov et al. (2022) utilized the YOLOv5m model for real-time monitoring and enhanced the detection accuracy of indoor fire disasters. In their study, Sreemathy et al. (2022) utilized four distinct deep learning models, namely, AlexNet, GoogleNet, VGG-16, and VGG-19 and Histogram Oriented Gradient (HOG) features for feature extraction for the automatic recognition of two-handed signs of Indian Sign Language. Montanha et al. (2022) employed a signal trilateration technique complemented by deep learning (DL) for image processing. Montanha et al. (2022) also leveraged a pre-trained deep neural network model from the open-source toolkit OpenVINO2 (Open Visual Inference and Neural Network Optimization) by Intel. Campomanes-Alvarez and Rosario Campomanes-Alvarez (2021) employed Long-Short Term Memory (LSTM) Neural Networks for jaw movement classification. Wadhwa et al. (2021) employed AI techniques involving preprocessing of raw image and caption data, using a pretrained Convolutional Neural Network (CNN) for encoding image features into high-dimensional vectors, and subsequently utilizing a Long Short-Term Memory (LSTM) based Recurrent Neural Network (RNN) for decoding and generating natural language descriptions. Lo Valvo et al. (2021) utilized Convolutional Neural Networks (CNNs) that have been trained to recognize objects or buildings. Guo et al. (2021) leveraged deep-learning technology for facial restoration, incorporated affective computing for emotion recognition, and employed these elements in the design of virtual avatars. Karyono et al. (2020) utilized artificial neural networks in their adaptive lighting system which considers behavioral adaptation aspects for visually impaired people. Lu et al. (2020) employed a tractor driving control method that combined EEG-based input with a recurrent neural network with transfer learning (RNN-TL) deep learning algorithm.

##### 4.3.2.6 More AI methodologies

Ingavelez-Guerra et al. (2022) utilized various AI techniques to adapt digital educational resources for the diverse needs of learners. It employs automatic speech recognition (ASR) to extract textual information from videos, thereby making the text more readable. In addition, the system leverages convolutional neural networks (CNNs) and recurrent neural networks (RNNs) for image classification and description. Furthermore, it utilized long short-term memory neural networks (LSTM NNs) from the Tesseract OCR library for text recognition. Natural language processing (NLP) has also been applied to aid in describing images using nearby text. Li et al. (2022) introduced a visual-tactile fusion classification model, a multimodal deep learning approach that combined visual and tactile information for improved object classification. Cimolino et al. (2021) applied AI techniques utilizing the Unity game engine along with the Behavior Designer plugin. The system developed by Kose and Vasant (2020) employs various AI techniques, including optimization-based AI techniques, such as the Dijkstra algorithm, ant colony optimization, intelligent water drop algorithm, and speech recognition interface. Watters et al. (2020) integrated an Alexa smart speaker and custom Alexa Skill for natural language interaction, a Talking LabQuest with AI for data collection and analysis, and a Raspberry Pi for coordination, effectively combining AI techniques with components to create a virtual AI lab assistant for enhanced laboratory assistance. In a study by Lin et al. (2019), machine learning and deep learning techniques, particularly neural networks, were harnessed for image recognition using YOLOv3, a real-time image recognition model, for object recognition and notification. In addition, chatbot technology was implemented using the LINE platform, demonstrating the integration of AI methodologies in both image recognition and chatbot development. Alashkar et al. (2020) employed a modern Convolutional Neural Network (CNN) named Mini Xception for facial features recognition, utilized the K-Nearest Neighbors (K-NN) algorithm for color recognition, and provided sound feedback using the IBM Watson Text to Speech API. These studies demonstrated the potential of combining deep learning with other techniques to develop more robust and adaptive systems that cater to diverse user requirements.

#### 4.3.3 (RQ 3) Dimension: challenges

A multitude of challenges confront efforts to enhance accessibility for people with disabilities by introducing barriers that may lead to exclusion and marginalization. Understanding and categorizing the various factors that influence the adoption of AI can aid stakeholders in devising specialized approaches to overcome the multifaceted challenges associated with its implementation. These challenges span various aspects, each of which requires careful consideration for successful implementation of accessible technologies.

##### 4.3.3.1 General accessibility and usability challenges

System complexity and lack of user friendliness can create difficulties for people with various disabilities, hindering their ability to use and benefit from technology. Implementation barriers, such as cost, lack of technical support, and insufficient awareness and training among users and caregivers, affect the overall accessibility and usability of AI systems (Lu et al., 2020; Cimolino et al., 2021; Theodorou et al., 2021).

##### 4.3.3.2 Data challenges

The incorporation of AI into digital accessibility systems has resulted in several data challenges. These challenges encompass matters pertaining to the inadequacy and questionable quality of data, along with apprehensions regarding data privacy, surveillance, administration, and the potential for bias and discrimination in the utilization of data within AI systems. The limited availability of training data, with people with disability comprising only 0.5–3% of the total user population, presents a significant challenge in achieving high levels of accuracy in speech recognition technology (Yang et al., 2022). Li et al. (2022) faced challenges in accurate tactile data acquisition for wearable devices, handling heterogeneous data from tactile and visual sensors. In their investigation, Zaid alahmadi and Alsulami (2020) encountered biases in the data, as the evaluation results of the prototype may have been influenced by the participation of only female participants, which overlooked the gender diversity present in Saudi Arabian universities. Additionally, Lu et al. (2020) encountered limitations in obtaining a comprehensive dataset because of constraints such as site-specific conditions, environmental variables, and tractor operating factors. Furthermore, maintaining consistency in virtual datasets is problematic, requiring ongoing algorithm adjustments (Lu et al., 2020). Acosta-Vargas et al. (2022) emphasized the importance of inclusive data collection processes, acknowledging the varying challenges faced by participants of different types and prominence of disabilities. This includes physical disabilities affecting phone usage, blindness posing challenges in photography tasks, considerations for cultural aspects and sign language fluency for deaf participants. These challenges highlight the need for innovative solutions and strategies to address the issue of data scarcity for specific user groups.

##### 4.3.3.3 Security and privacy challenges

Considering the privacy and security challenges associated with integrating assistive technologies, several issues have been noted in various studies. The accurate acquisition of tactile data for wearable devices raises privacy and security concerns, particularly related to webcam usage (Li et al., 2022). Similarly, the development of digitally accessible games introduced ethical dilemmas, encompassing issues of player autonomy, privacy, and potential unintended consequences tied to partial automation. Privacy and security concerns were also integral to the challenges faced in designing an indoor navigation system (Lo Valvo et al., 2021) and developing the avatar-to-person system (Guo et al., 2021), emphasizing the necessity for technical expertise in AI and 3D modeling. The study conducted by Zaid alahmadi and Alsulami (2020) faced challenges linked to the need to navigate privacy concerns, especially given the impact of including only female participants. Involving people with disabilities in data collection processes underscores the significance of inclusive practices, recognizing diverse accessibility challenges and implicitly emphasizing the importance of safeguarding privacy (Acosta-Vargas et al., 2022). These challenges collectively highlight the multifaceted nature of integrating assistive technologies and the imperative to address privacy and security across various domains.

##### 4.3.3.4 Challenges of visually impaired

Karyono et al. (2020) drew attention to the deficiency of a dependable predictive lighting model for visually impaired and elderly individuals, the scarcity of implementation of lighting standards, and potential barriers such as system cost and insufficient awareness. Similarly, Jayawardena et al. (2019) emphasized the challenges in enhancing the lives of visually impaired children, including the dearth of research studies, limited resources and funding, and the requirement for user-friendly, affordable systems in developing countries.

##### 4.3.3.5 Challenges of hearing impaired

The challenges faced by Teófilo et al. (2018) include technical setup issues affecting user experience, accuracy of captioning for users with hearing impairments, and usability factors such as head and eye strain, brightness, and device weight.

##### 4.3.3.6 Challenges faced by those with motor disabilities

Li et al. (2022) presented several challenges in the acquisition of accurate tactile data for wearable devices, including the handling of heterogeneous data and concerns related to privacy and security, which can impact users with varying motor abilities. Additionally, Cimolino et al. (2021) highlighted challenges in the development of digitally accessible games, particularly in interpreting the intentions of players with diverse motor abilities and designing interfaces for partial automation.

##### 4.3.3.7 Challenges faced by those with cognitive disabilities

Park et al. (2021) involved people with disabilities and highlighted the challenges faced by those with cognitive disabilities, such as difficulties with reading and typing tasks, including people with attention deficit hyperactivity disorder (ADHD) and dyslexia.

##### 4.3.3.8 Other challenges

Other challenges include the lack of resources and funding for the research and development of systems that enhance accessibility (Jayawardena et al., 2019). Lo Valvo et al. (2021) faced challenges related to physical infrastructure adaptation, 3D object registration, and accessibility design. Guo et al. (2021) identified challenges related to psychological barriers, accessibility issues, the need for technical expertise in relevant areas, and the importance of user acceptance and adoption influenced by factors such as ease of use and perceived usefulness.

#### 4.3.4 (RQ 4) Dimension: frameworks

Various frameworks and accessibility standards have been employed in AI-based research endeavors aimed at enhancing digital accessibility for people with disabilities. One notable framework is the “Framework for value co-design and co-creation” (Vieira et al., 2022), which centers on the impact of Virtual Assistants (VAs) on the wellbeing of people with disabilities. This framework emphasizes the co-creation of value through interactions between people with disabilities and VA technology within their home environments.

Additionally, addressing the adaptability and accessibility of Open Educational Resources (OER), the need for new metadata aligned with Universal Design for Learning guidelines is highlighted (Ingavelez-Guerra et al., 2022). Cimolino et al. (2021) explored the improvement in game accessibility by utilizing multiple frameworks, including universal design, player balancing, and interface adaptation. Within this context, universal design focuses on the creation of products and environments that cater to a broad spectrum of individuals irrespective of their abilities.

In the domain of e-commerce website accessibility, Acosta-Vargas et al. (2022) predominantly relied on the Web Content Accessibility Guidelines (WCAG) 2.1, employing a modified approach to the Website Accessibility Conformance Evaluation Methodology (WCAG-EM) 1.0, for automated evaluations using the Web Accessibility Evaluation Tool (WAVE) to identify potential WCAG 2.1-related accessibility concerns. Furthermore, See and Advincula (2021) evaluated system usability through the ISO/IEC 25010:2011 SQuaRE quality models, which encompass factors such as appropriateness, recognizability, learnability, operability, user error protection, user interface aesthetics, and accessibility.

The Digital Accessible Information System (DAISY) standard allows for utmost flexibility in integrating text and audio, accommodating various combinations ranging from pure audio, text-only, full text, to full audio integration.

Karyono et al. (2020) employed various accessibility standards, including CIE 1,231:1997 for addressing lighting needs of partially sighted individuals, CIE 196:2011 for enhancing accessibility in lighting, and CIE 227:2017 for lighting considerations in buildings for older individuals and those with visual impairments.

In their study, Hezam et al. (2023) harnessed a decision-making framework grounded in Fermatean fuzzy information, incorporating AI methodologies from the field of expert systems, to comprehensively evaluate the suitability of digital technologies within the realm of sustainable transportation for people with disabilities.

Yeratziotis and Van Greunen (2013) outlined guidelines for deaf and hard of hearing-friendly mobile app design, drawing from sources like telecom accessibility guidelines and industry practices (e.g., Apple, Samsung, and Google) which were used to guide the system developed in Zaid alahmadi and Alsulami (2020).

Table 7 shows the results of the classification framework.

**Table 7:** Mapping the classification framework across the different disabilities.

	**Visual impairment**	**Speech and hearing impairment**	**Autism spectrum disorder (ASD)**	**Neurological disorders**	**Motor impairment**
AI methodologies		Edge AI			
	Computer vision				
	Machine learning				
	Deep learning				
	NLP				
AI accessibility/standard/ framework	Framework for value co-design and co-creation				
	Web Content Accessibility Guidelines (WCAG) 2.1				
	Universal Design				
		Guidelines for designing mobile apps for people who are deaf or hard of hearing (Yeratziotis and Van Greunen, 2013)			
	• Digital Accessible Information System (DAISY) Standard • Low Vision—Lighting needs for the partially sighted: CIE 1,231,997; CIE 196:2011; CIE 227:2017				
Challenges	Data challenges				
	Technical issues				
	Operational challenges				
	Knowledge and awareness				
	Security and privacy		Security and privacy		Security and privacy
Applications	• Artificial vision • Navigation • Virtual Assistant • Fire Safety • Road Safety • Educational Material Generation • Social Media accessibility • Bank Note Recognition • Lighting system • Walking path support • Image captioning	• Speech recognition • Voice reproduction • Communicating with smart devices • Video transcription • Harness VR technology for communication during theatrical performance	Learning to code	Accessibility in learning	• Enhance digital games accessibility • Accessible bus rides • Traffic signal assistance
		Sign language recognition			

### 4.4 Research implications, limitations, and future directions

Most research in the field of AI-driven digital accessibility has primarily focused on addressing the needs of people with visual impairment. This emphasis on AI solutions related to visual impairment is evident from the multitude of innovations and systems designed to enhance the lives of the visually impaired. These include a wide range of applications, from real-time obstacle detection and object recognition to tactile educational materials, and even AI-driven smart glasses. The impact of AI on digital accessibility for the visually impaired has been profound, leading to improved mobility, safety, and educational opportunities (Lo Valvo et al., 2021; See and Advincula, 2021; Abdusalomov et al., 2022). However, this concentration of visual impairments has highlighted a significant gap in the research landscape. There is a paucity of comprehensive AI systems tailored to address the unique challenges faced by people with other disabilities such as speech and hearing impairments, autism spectrum disorder (ASD), neurological disorders, and motor impairments. While there are some noteworthy AI solutions for these other disability types (Zingoni et al., 2021; Ullah et al., 2023), the sheer volume of research and innovation predominantly dedicated to visual impairment underscores the need for a more equitable distribution of research efforts. It is crucial to expand the scope of AI-driven digital accessibility to bridge this gap and provide people with various disabilities with the same level of support, independence, and accessibility that the visually impaired enjoy. This will require concerted effort to foster innovation and research in AI systems tailored to the specific needs of these communities. Our research highlights the urgent need for a fundamental shift in the design and development of systems catering to people with disabilities.

To address these shortcomings, the research agenda must prioritize a 2-fold approach. First, researchers must realign their efforts toward a more comprehensive examination of disabilities, ensuring that the unique challenges faced by people with diverse impairments are adequately considered. Second, it is imperative to augment data-collection efforts involving people with disabilities to capture a broader range of experiences and requirements. This inclusive approach not only enhances the inclusivity of technological solutions but also provides a more robust and informed foundation for future research and development in the field. A more focused effort is necessary to comprehend how the information requirements of people with disabilities can vary across different contexts, cultures, and audiences and how their needs are context-dependent (Akter et al., 2022). Future research should explore this aspect to enhance the AI-driven accessibility. Park et al. (2021) suggested that motivating people with disabilities for AI data collection should involve fair monetary compensation, non-monetary incentives, and transparent communication regarding data use and privacy. To ensure accessibility, the data collection process should be streamlined and consider the diverse range of abilities within disability categories, avoid punitive measures, and acknowledge potential performance anxiety among people with disabilities. Future research should prioritize algorithmic accountability, transparency, and explainability in the development of assistive technologies (Akter et al., 2022). This approach ensures that users can better understand and trust the functioning of AI-driven systems, thereby contributing to their overall effectiveness and acceptance. Maintaining the confidentiality and privacy of the collected data is crucial, especially when the data are publicly available to promote large-scale machine learning advancements (Li et al., 2022). It is essential to extend the reach of the infrastructure to individuals beyond WEIRD societies, who may use different platforms, devices with lower specifications, or have limited access to broadband, and to support cultural adaptations in the data collection process (Li et al., 2022).

Although every effort has been made to ensure the comprehensiveness of the present research, it is possible that some significant studies may have been overlooked because of the vast number of results retrieved from one of the databases.

## 5 Conclusion

The significance of access to the Internet and digital content has intensified in recent times; however, not all individuals, particularly those with disabilities, have equitable access to this technology. By employing a thorough review and evaluation using a classification framework, this research specifically focuses on the impact of AI on digital accessibility in the context of disability. The most prevalent AI methodologies utilized are edge AI, NLP, Computer Vision, machine learning, and deep learning. The most common challenges encountered were related to data, technical difficulties, security and privacy concerns, and operational difficulties. The findings reveal a prevalent focus on AI-driven digital accessibility for people with visual impairments, indicating a substantial gap in addressing other disabilities. This research underscores the imperative need to realign efforts toward a more comprehensive examination of disabilities, urging researchers to broaden their scope and enhance data collection efforts involving people with various disabilities. The shortcomings of existing systems regarding adherence to accessibility standards highlight the pressing need for a fundamental shift in the design of solutions that prioritize the needs of people with disabilities. The study underscores the critical role of accessible AI in preventing exclusion and discrimination and emphasizes the urgency for a comprehensive approach to digital accessibility that accommodates diverse disability needs. As we move forward into the digital age, where Internet access is increasingly integral to education, entertainment, and communication, organizations are encouraged to prioritize and invest in digital accessibility. By adhering to established guidelines and standards, organizations can bridge the digital divide and ensure a fair and enjoyable online experience for all users regardless of their abilities. This not only promotes equal opportunities for individuals with disabilities, but also enhances overall usability and satisfaction for all users. Ultimately, this research calls for a concerted effort to make digital accessibility a cornerstone of our digital landscape, fostering an inclusive environment that benefits the entire user spectrum.

## Data availability statement

The raw data supporting the conclusions of this article will be made available by the authors, without undue reservation.

## Author contributions

KC: Data curation, Investigation, Methodology, Resources, Software, Visualization, Writing – original draft, Writing – review & editing. AO: Conceptualization, Methodology, Project administration, Supervision, Validation, Writing – original draft, Writing – review & editing.
